# Reflections on my 15 years as *mBio* editor in chief

**DOI:** 10.1128/mbio.01741-25

**Published:** 2025-06-24

**Authors:** Arturo Casadevall

**Affiliations:** 1 Johns Hopkins Bloomberg School of Public Health 25802 , Baltimore, Maryland, USA

## EDITORIAL

As my time as *mBio* editor in chief concludes on 30 June this year after leading the journal for more than 15 years, I revisit *mBio*’s journey and highlight key accomplishments.

*mBio* was born in Philadelphia in May 2009. There, while attending the old American Society for Microbiology (ASM) general meeting, I received an email from Dr. Thomas Shenk saying he wanted to talk to me. Dr. Shenk was chair of the ASM Journals board at the time. I had no inkling about the topic of the meeting, but I knew Dr. Shenk and I agreed. The meeting occurred in a hotel suite, and I remember that food had been ordered, including a bottle of wine. In attendance were Drs. John Collier, former chair of the American Academy of Microbiology (AAM) Board of Governors, and Barbara Goldman, who was the director of ASM Journals at the time.

After some pleasantries, Dr. Shenk got right to the point. ASM wanted to launch its first open-access journal, and it was to be selective, publishing only the best microbiology research. Dr. Collier was at the meeting because Dr. Shenk had already anticipated involving the AAM in the new journal. Minutes into our meeting, I agreed to head the new journal. Next came a discussion about the mechanics of launching an open-access journal, something that was still somewhat of a novelty at the time and ASM had never done before. An hour or so into our meeting, Dr. Shenk opened the bottle of wine to celebrate the new enterprise.

One early critical decision was to create a broad-scope journal focusing on all areas of microbiology and its allied sciences. By allied sciences, we meant disciplines that were important for some fields of microbiology, such as immunology. That goal appealed to me since I consider myself a generalist with broad interests in microbiology, immunology, and all the sciences. ASM had no experience with broad-scope journals since it had, over decades, created a stable of successful specialty journals such as *Journal of Bacteriology* and *Journal of Virology*. Creating and running a broad-scope journal is more difficult because success means having the editorial expertise to handle manuscript submissions on diverse topics. *mBio* solved that problem by creating a large board of editors and relying on invited editors when necessary. Using that model, ASM eventually launched two other broad-scope, open-access journals, *mSphere* and *Microbiology Spectrum*.

Within days after the Philadelphia meeting, Dr. Goldman and I began to work on the new journal. Shortly thereafter, Maisha Miles was hired as managing editor, and Rob Arthur joined the journal as assistant managing editor. The group had great personal chemistry, and I do not remember a single disagreement. Consequently, in those early days, things moved rapidly. Dr. Goldman, Ms. Miles, and I, with input from Dr. Shenk, made innumerable rapid decisions such that by the winter of 2009–2010, *mBio* was ready to receive papers. The first submission came from a conversation with Dr. Peter Palese at a 2010 New Year’s party. Dr. Palese mentioned to me that he had an important paper showing that the conserved hemagglutinin domain of influenza virus could be used as a vaccine and that the paper had run into turbulence at some high-end journals. I immediately asked him to consider *mBio* and promised him a rapid review and decision. Dr. Palese is an eminent virologist, and having a paper from his laboratory would signal to others that it was a worthy place to publish. He submitted it to the journal in January, and that paper was the first paper in *mBio* (1). By late spring 2010, we had sufficient papers to produce the first issue of *mBio*, and the journal was off and running (https://journals.asm.org/toc/mBio/1/1). If I add the year between inception in 2009 and launching in 2010, then my time with *mBio* is not a quindecennial, but rather 16 years.

A major innovation with the launching of *mBio* was the AAM track, whereby academy members could submit one paper per year with reviews from peers in the field (2). The AAM track was modeled on the relationship between *Proceedings of the National Academy of Sciences* (*PNAS*) and the United States National Academy of Sciences, where academy members could arrange for the review of their papers before communicating them to that journal. In the early days of the journal, the AAM track contributions constituted a significant proportion of manuscripts published, thus providing the journal with a steady stream of excellent publications. In 2011, AAM contributions constituted a third of all submissions, but by 2024, this number was down to 5%. Hence, a case can be made that the AAM contributed disproportionately to the success of *mBio* during the early vulnerable years of its existence. The AAM track also benefited the academy since the launching of the *mBio* privilege for academy members was temporally associated with a large increase in nominations for election to fellowship.

As I reflect on the success of *mBio*, it is clear to me that the lion’s share of credit must go to the editors and the journal staff, for it is they who make the journal work. Running a successful journal means managing a complex set of moving parts, which include a constant stream of incoming papers, papers under review, papers in the press, tracking the status of reviews, handling queries from authors, and ensuring compliance with journal rules. Keeping the moving parts in working order is the job of the staff, and *mBio* has been blessed with the most dedicated and outstanding staff who have been the heart and soul of our journal. *mBio* was successful in attracting an exceptional cadre of editors who included some of the most prominent scientists in the field. They, in turn, made the decisions on what to publish, and these decisions, in aggregate, gave content to the journal.

Apart from a quindecennial of publishing, top science *mBio* also provided a venue to air issues of importance to science. During the great gain-of-function debates of 2012–2014, *mBio* provided a venue for airing various viewpoints. That was a time when much of the debate was being conducted in public, with scientists providing their opinions to reporters, and we felt that it was important to get the arguments for and against such experimentation written down and developed in the context of scientific publication. *mBio* also took on the mission of training the next generation of editors by creating the Early Career Editorial Board, where early career scientists are taught the nuts and bolts of how reviews and decisions are done (3). The Early Career Editorial Board was the brainchild of Ms. Maisha Miles and is an example of the expanding roles of journals as they take on tasks to support the scientific enterprise that transcend their traditional roles as venues for the publication of new information.

I know that the transition ahead will be smooth. Dr. Marvin Whiteley will be the next editor in chief. He is a highly accomplished microbiologist with a broad vision who has been my friend for more than a decade. Dr. Whiteley will be aided by a group of highly dedicated people who manage day-to-day journal operations and coordinate with ASM leadership and other ASM journals. They include Miriam Day (assistant managing editor), Noel Lin (managing editor), Lorraine Clark (journals development editor), Nicole Glenn (senior associate, peer review), and Becky Zwadyk (production editor). This outstanding group has already planned for a smooth transition. On a personal note, I will miss working with them and assure them that I am here if I can be of help in the future.

In 2025, *mBio* is thriving, with a large number of submissions projected for this year. The number of submissions is an important parameter of journal health because it is an indicator of how it is perceived by authors, and with more submissions, there is a greater probability for more great papers. For me, it was a great honor and privilege to witness the birth of the journal and its growth into a major journal in the field of microbiology and its allied sciences. However, I am not leaving *mBio*. I’m staying on as editor for the Story behind the Science series, which focuses on reporting details and stories about the functioning of science that rarely make their way into publications (4).

## References

[1] Steel J, Lowen AC, Wang TT, Yondola M, Gao Q, Haye K, García-Sastre A, Palese P. 2010. Influenza virus vaccine based on the conserved hemagglutinin stalk domain. mBio 1:e00018-10. doi:10.1128/mBio.00018-10 20689752 PMC2912658

[2] Casadevall A, Collier RJ. 2011. Peer review of manuscripts submitted via the Academy Fellowship track. mBio 2:e00075-11. doi:10.1128/mBio.00075-11

[3] Casadevall A, Miles M, Day M, Donaldson A. 2024. The mBio Early-Career Editorial Board Reviewer Training Program: cultivating excellence in peer review. mBio 15:e01763-24. doi:10.1128/mbio.01763-24 38874409 PMC11253583

[4] Casadevall A, Clark LF. 2025. The Story behind the Science: shining a light on the path to discovery. mBio 16:e03679-24. doi:10.1128/mbio.03679-24 39835805 PMC11898762

